# Expression of artemin and GFRα3 in an animal model of migraine: possible role in the pathogenesis of this disorder

**DOI:** 10.1186/s10194-016-0673-2

**Published:** 2016-09-06

**Authors:** Hai-Qiong Shang, Yan Wang, Yan-Yan Mao, Li-Gang Kong, Gao-Ying Sun, Lei Xu, Dao-Gong Zhang, Yue-Chen Han, Jian-Feng Li, Hai-Bo Wang, Zhao-Min Fan

**Affiliations:** 1 Department of Otolaryngology-Head and Neck Surgery, Shandong Provincial Hospital Affiliated to Shandong University, Jinan, 250021 People’s Republic of China; 2 Shandong Provincial Key Laboratory of Otology, Jinan, 250022 People’s Republic of China; 3 Institute of Eye and ENT, Shandong Provincial Hospital Affiliated to Shandong University, Jinan, 250022 People’s Republic of China; 4 Department of Otolaryngology, People’s Hospital of Rizhao, Rizhao, 276800 People’s Republic of China

**Keywords:** Migraine, Artemin, GFRα3, Dura mater, Trigeminal ganglia

## Abstract

**Background:**

Neurotrophic factors have been implicated in hyperalgesia and peripheral levels of these molecules are altered in migraine pathophysiology. Artemin, a vasculature-derived neurotrophic factor, contributes to pain modulation and trigeminal primary afferent sensitization through binding its selective receptor GFRα3. The distribution of artemin and GFRα3 in the dura mater raises an anatomy supports that they may be involved in migraine. In this study we evaluated the expression of artemin and GFRα3 in an animal migraine model that may be relevant for migraine.

**Methods:**

In this study, using a rat migraine model by administration of nitroglycerin (NTG), we investigated the expression of artemin in the dura mater and GFRα3 in the trigeminal ganglia (TG) by means of quantitative reverse transcription-polymerase chain reaction, western blot and immunofluorescence labeling.

**Results:**

Artemin immunoreactivity was found in the smooth muscle cells of dural vasculature and GFRα3 was present in cytoplasm of TG neurons. The mRNA levels of artemin and GFRα3 were significantly elevated after NTG treatment at 2 and 4 h respectively (*P* < 0.05). The expression of artemin protein was increased at 4 h and continually up to 8 h in the dura mater following NTG administration (*P* < 0.05). The expression of GFRα3 protein was elevated at 4 h and continually up to 10 h in the TG following NTG administration (*P* < 0.05).

**Conclusion:**

The findings suggest that artemin and GFRα3 play an important role in the pathogenesis of migraine and may represent potential therapeutic targets for the treatment of migraine.

## Background

Migraine is a common neurovascular disorder characterized by recurrent attacks of typically throbbing and unilateral headache, which affects up to 20 % of the population [1]. However, its pathophysiology has not yet been fully elucidated. To date, on the basis of clinical observations and experimental studies, several theories have been proposed for the mechanisms underlying this disorder. In particular, one theory, which focuses on the trigeminovascular system, has been widely accepted in migraine pathogenesis [2, 3]. Many studies indicate that activation of peripheral trigeminal nociceptors in the dura mater results in the release of neuropeptides and neurotrophins, which are related to the generation and modulation of migraine pain [4, 5].

In recent years, artemin, a member of the glial cell line-derived neurotrophic factor (GDNF) family, has aroused considerable interest because it not only modulates the development and function of sensory neurons [6, 7], but also participates in the pathophysiology of peripheral inflammation and pain hyperalgesia [8]. Artemin exerts its influence on intracellular signaling pathways via binding to the receptor complex of GDNF family receptor alpha 3 (GFRα3) and ret [9]. GFRα3, a highly selective receptor for artemin, is co-expressed with the transient receptor potential vanilloid 1 (TRPV1) in the dorsal root ganglia (DRG) and trigeminal ganglia (TG) [10, 11]. TRPV1, a calcium permeable ion channel, is activated by heat and capsaicin, and is considered to play a major role in the pathogenesis of migraine [12, 13]. In addition, the detection of the distribution of artemin and its receptor GFRα3 in the dura mater of rats suggests that artemin may contribute to migraine pain by the sensitization of dural afferents [11].

It is known that systemic administration of nitroglycerin (NTG), a nitric oxide (NO) donor, can induce delayed headaches in both migraneurs and healthy people [14], and NTG-treated rodents have been found to be predictive animal models of migraine [15]. Previous reports have shown that the rat migraine model triggered by NTG present vasodilation of the meningeal vessels and lead to the release of proinflammatory substances [16]. In this regard, the present study was designed to evaluate the expression of artemin in the dura mater and GFRα3 in the TG following NTG administration, so as to explore the possible mechanisms of artemin and GFRα3 underlying the pathogenesis of migraine.

## Methods

Male wistar rats (weight 260–300 g) were purchased from Animal Centre of Shandong University (Jinan, China) and maintained in a humidity-controlled as well as thermoregulated vivarium with a 12-h light/dark cycle. The animal care and experimental protocol were approved by the Animal Care Committee of Shandong University, PR China (NO. ECAESDUSM 20123011).

Eighty wistar rats were stochastically divided into three groups: normal control, normal saline (NS) control, and the NTG groups. NTG at dose of 10 mg/kg was injected cervical subcutaneously to set up the rat model of migraine [17]. In the NS group, rats were treated with isotonic saline using the same volume as NTG. At different time points after NTG or NS treatment, rats were anaesthetized with 10 % chloral hydrate (4 μl/g). Then the temporal bones were removed and the dura was carefully dissected out. After removing the brain halves, the TG tissues were separated from the cranial base. Some tissue samples were quickly frozen at −80 °C for further western blot analysis and qRT-PCR, others were fixed in 4 % paraformaldehyde for immunofluorescent staining.

### Quantitative reverse transcription-polymerase chain reaction (qRT-PCR)

Total RNA was extracted from the dura mater and TG using the Trizol Reagent (Invitrogen, Gaithersburg, USA) according to the manufacturer’s instructions. For each sample, 1 μg total RNA was reverse transcribed using the ExScript RT reagent kit (TaKaRa, Dalian, China). Using SYBR Green PCR kits, qPCR was performed on an Eppendorf AG 22331 Hamburg machine (Germany). Forward and reverse primer sequences applied in real-time PCR were as follows: artemin-F: 5′-ACTCATTCCTGGTTGCCTTCT-3′; artemin-R: 5′-GGTCTTCACCTTCCATTCAGA-3′; GFRα3-F: 5′-ACTCATTCCTGGTTGCCTTCT-3′; GFRα3-R: 5′-GGTCTTCACCTTCCATTCAGA-3′; β-actin-F: 5′-GTGGGGCGCCCCAGGCACCA-3′; β-actin-R: 5′-CTCCTTAATGTCACGCACGATTT-3′; GAPDH-F: 5′-GTGGGGCGCCCCAGGCACCA-3′; GAPDH-R: 5′-CTCCTTAATGTCACGCACGATTT-3′. The calculated number of specific transcripts was normalized by β-actin or GAPDH. The real-time value for each sample was averaged and compared by using the CT method. The amount of target RNA (2^-ΔΔCt^) was normalized both with the endogenous β-actin or GAPDH reference (ΔCt) and theamount of target gene in control sample, which was set as the calibrator at 1.0.

### Western blot analysis

Total protein was extracted using radioimmune precipitation buffer protein lysis buffer according to protocols (Beyotime, Shanghai, China). The protein content of the samples was measured by means of the BCA protein assay kit (Beyotime, Shanghai). The protein samples were denatured and separated by 10 % sodium dodecyl sulfate-polyacrylamide gel electrophoresis and transferred onto nitrocellulose membranes. The nitrocellulose membranes were blocked for 1 h at room temperature in 5 % skimmed dried milk. Then the membranes were incubated with primary antibodies including: rabbit antibody to GFRα3 (1:1000, Abcam Systems, USA), rabbit antibody to artemin (1:200, Abbiotec, San Diego) and mouse antibody to β-actin (1:1000, Santa Cruz Biotechnology, USA) in TBST containing 3 % fat-free dry milk for 1 h at room temperature and overnight at 4 °C. After washing 3 times with TBST, the membranes were incubated with the secondary donkey anti-rabbit or anti-mouse IgG antibodies (1:5000, Santa Cruz, USA) at room temperature for 1 h. Finally, the immunoblots were detected using an ECL kit (Santa Cruz, USA) and visualized after exposure to X-ray films. The relative optical density ratio was calculated with the Image J software by comparison to GAPDH or β-actin.

### Immunofluorescence staining

Tissues were fixed in 4 % paraformaldehyde at 4 °C for 24 h, followed by incubated in 30 % sucrose for 24 h, embedded in optimum cutting temperature compound for 24 h, quickly frozen with liquid nitrogen, and cut into 6 μM sections using a cryostat (Leica CM 1850, Nussloch, Germany). For immunofluorescence, samples were washed in 0.01 M PBS and blocked in PBS containing 0.3 % Triton X-100 (Sigma, USA) and 10 % donkey serum (NQBB, USA) for 1 h at room temperature and incubated in primary antibodies overnight at 4 °C. The following antibodies were used: goat antibody to GFRα3 (1:50, R&D Systems, USA), rabbit antibody to artemin (1:50, Abbiotec, San Diego) and mouse antibody to α-smooth muscle actin (α-SMA) (1:50, Dako Cytomation, Germany), After being washed fully in PBS, samples were incubated with secondary antibodies (1:1000, Invitrogen, USA) and 49,6-diamidino-2-phenylindole (DAPI) (1:1000, Molecular Probes, USA) in PBS at room temperature for 1 h. Then rinsed in PBS for 30 min. Immunofluorescent samples were visualized with an inverted DMI 400CS confocal microscope (Leica, Germany).

### Statistical analysis

The statistical analyses were performed using SPSS 17.0 software (SPSS Inc., Chicago, ILL, USA). Data were expressed as mean ± standard error of the mean (SEM). One-way Analysis of variance (ANOVA) was used for statistical analysis and *P* < 0.05 was considered statistically significant.

## Results

### Expression of artemin in the dura mater

**Fig. 1:**
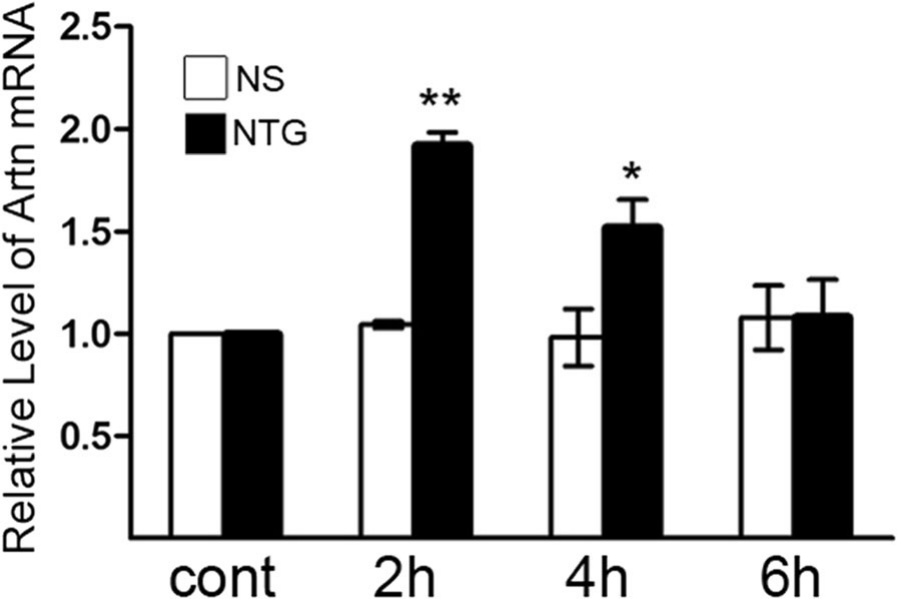
Alteration of artemin mRNA expression in the dura mater after NTG treatment. The expression of artemin (Artn) mRNA was significantly increased at 2 h, 4 h following NTG treatment compared with that in the normal control group (^**^ *P* < 0.01, ^*^ *P* < 0.05). No significant change in NS control group was observed compared to normal control group. The data shown here were mean ± SEM of three separate experiments

In consideration of NTG-induced delayed onset inflammation in migraine model rats, the mRNA expression of artemin in the dura mater was detected after NTG or NS administration for 2, 4 and 6 h each. As shown in Fig. 1, the mRNA level of artemin was significantly elevated at 2 and 4 h after NTG administration compared to the normal control group. There was no significant difference in artemin mRNA expression between the NS control group and the normal control group.

**Fig. 2:**
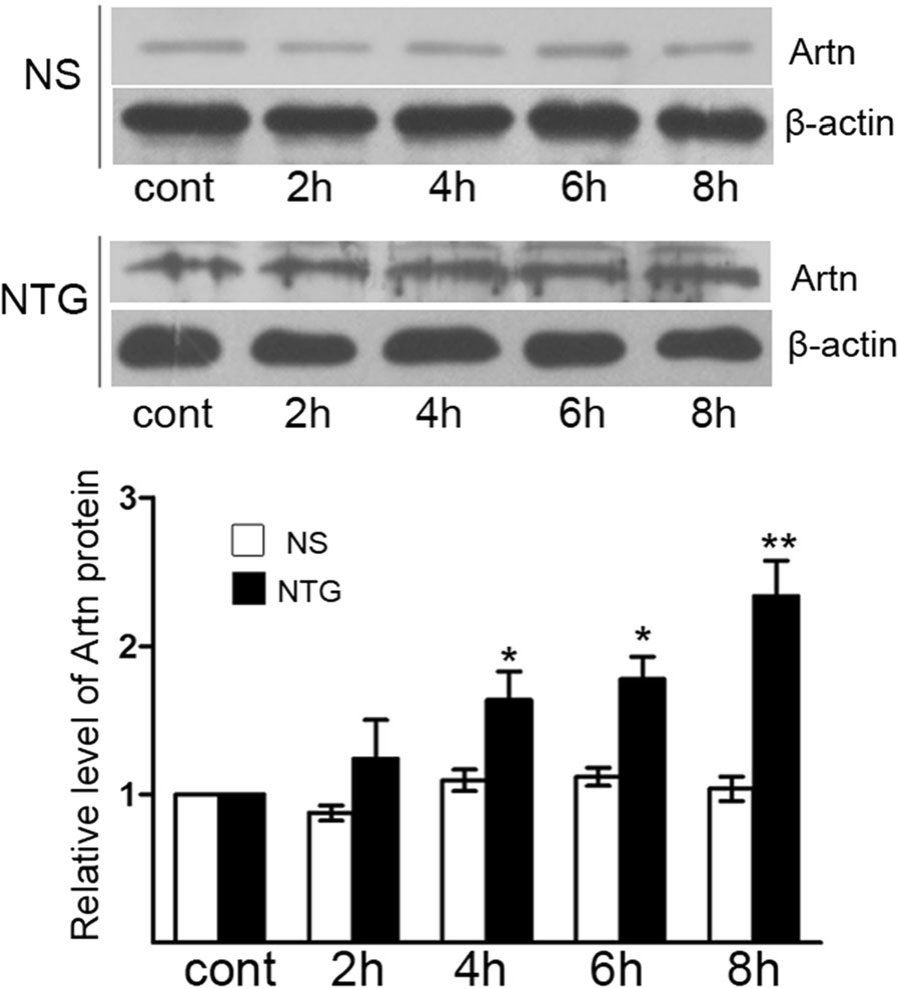
Increased protein level of artemin in the dura mater following NTG treatment. Western blots for artemin (Artn) and β-actin proteins in the dura mater after NTG treatment. The protein expression of Artn was dramatically elevated at 4, 6 and 8 h compared to normal control group (^**^ *P* < 0.01, ^*^ *P* < 0.05). There was no significant difference between NS control group and normal control group. The data shown here were mean ± SEM of three separate experiments

Four time points (2, 4, 6 and 8 h) were chosen to evaluate the protein level of artemin in the dura mater following NTG or NS injection. As shown in Fig. 2, the expression of artemin protein was low in both normal control group and NS control group. However, the protein expression of artemin in the dura of rats after NTG injection from 4 to 8 h was dramatically increased compared with that in the normal control rats.

### Expression of GFRα3 in the TG

**Fig. 3:**
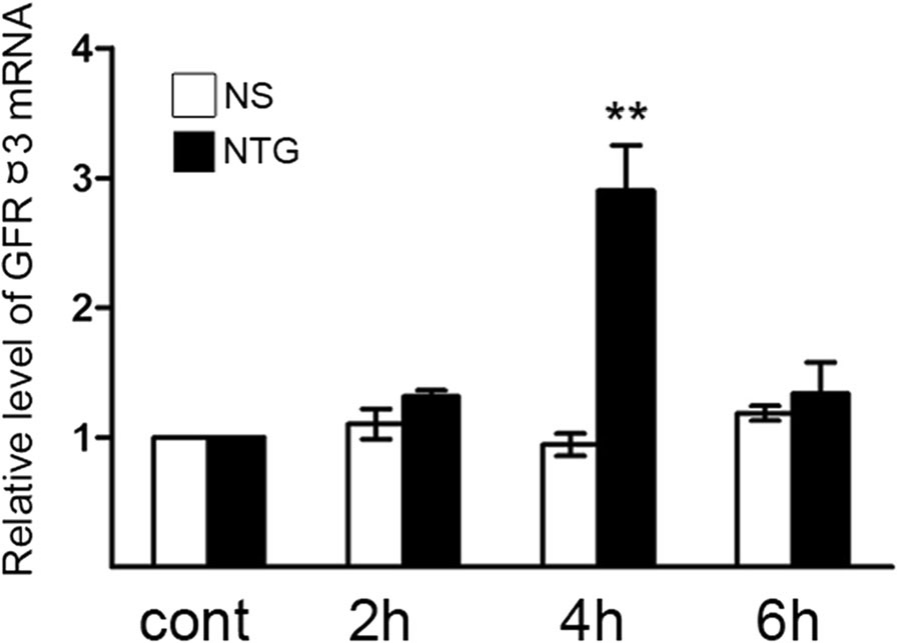
Alteration of GFRα3 mRNA expression in the TG after NTG treatment. The expression of GFRα3 mRNA was significantly increased at 4 h following NTG treatment compared with that in the normal control group (^**^ *P* < 0.01). No significant change in NS control group was observed compared to normal control group. The data shown here were mean ± SEM of three separate experiments

The mRNA expression of GFRα3 in the TG was detected after NTG or NS administration for 2, 4 and 6 h each. As shown in Fig. 3, the mRNA level of GFRα3 in the TG was markedly increased at the 4-h time point after NTG administration compared to the normal control group. There was no significant difference in GFRα3 mRNA expression between the NS control group and the normal control group.

**Fig. 4:**
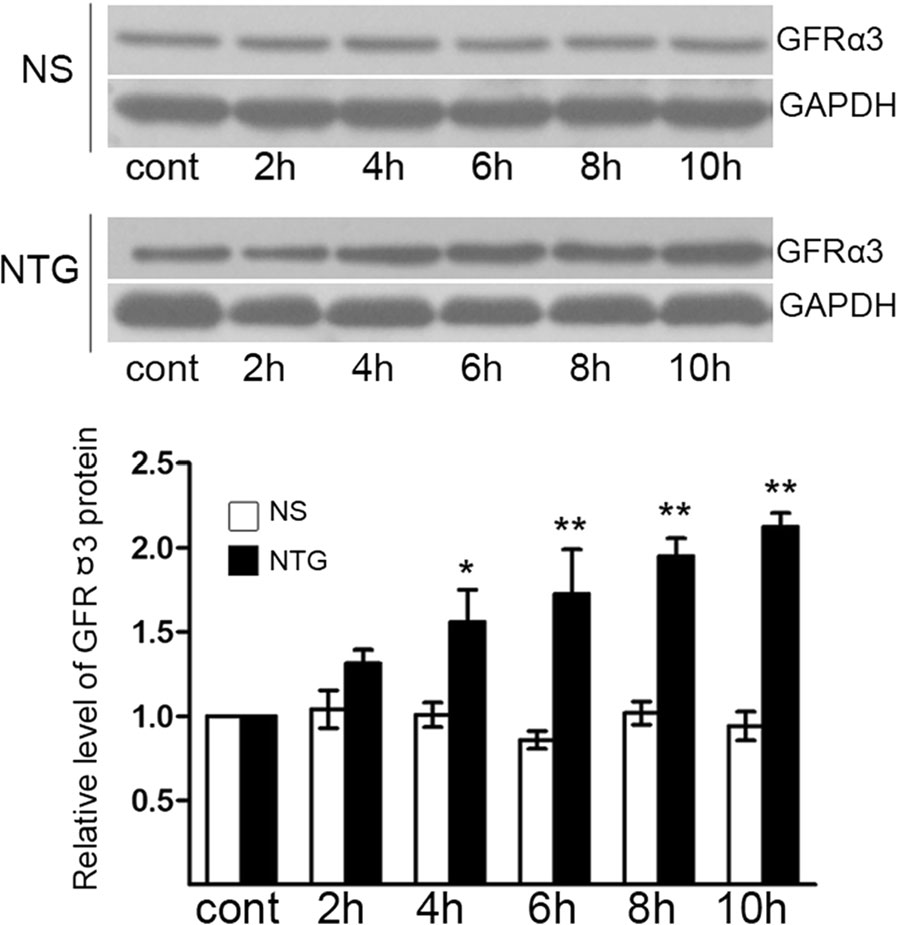
Increased protein level of GFRα3 in the TG following NTG treatment. Western blots for GFRα3 and GAPDH proteins in the TG after NTG treatment. The protein expression of GFRα3 was dramatically elevated at 4 h and continually up to 10 h compared to normal control group (^**^ *P* < 0.01, ^*^ *P* < 0.05). There was no significant difference between NS control group and normal control group. The data shown here were mean ± SEM of three separate experiments

Five time points (2, 4, 6, 8, and 10 h) were chosen to evaluate the protein level of GFRα3 in the TG after NTG or NS injection. As shown in Fig. 4, the expression of GFRα3 protein was low in both normal control group and NS control group. However, the protein expression of GFRα3 in the TG after NTG injection from 4 to 10 h was markedly increased compared with that in the normal control rats.

### Immunofluorescence labeling for artemin and GFRα3

**Fig. 5:**
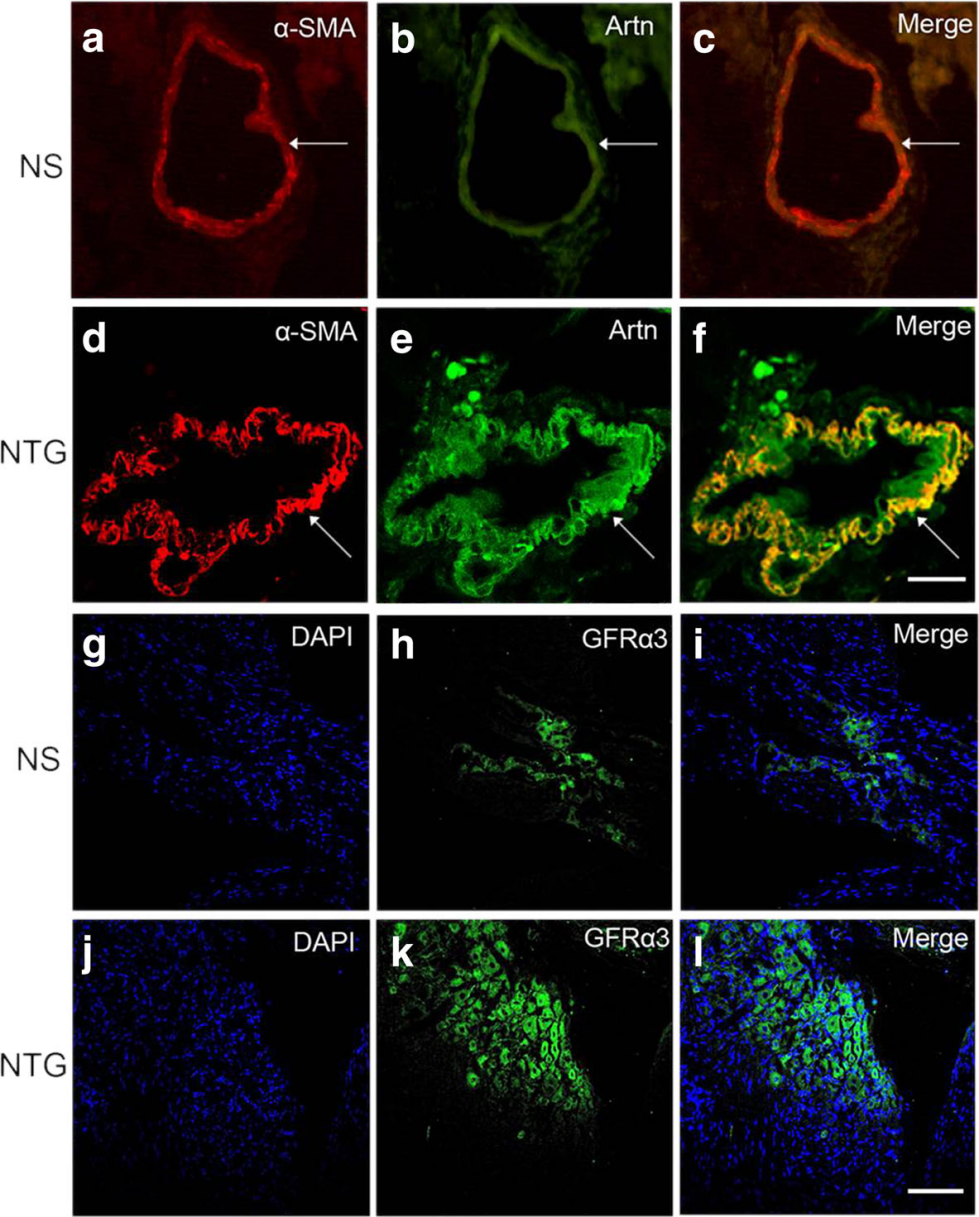
Immunostaining of artemin in the dura mater and GFRα3 in the TG after NTG treatment. **a**-**c** Immunohistochemical staining for α-SMA (*red*) and artemin (*Artn*, *green*) in the dura mater of NS control group. **d**-**f** Artemin was strongly expressed in the dura mater after NTG treatment. **g**-**i** Immunohistochemical staining for DAPI (*blue*) and GFRα3 (*green*) in the TG of NS control group. **j**-**l** The number of GFRα3-positive neurons was elevated after NTG treatment. **c**, **f**, **i**, **l** The same field of merged vision. Scale bar = 100 μm

Double immunofluorescence labeling proved the co-localization of artemin and α- smooth muscle actin (α-SMA), a maker for smooth muscle cells, on the dural blood vessels. Additionally, the immune positive signal of artemin that it was observed after NTG was obviously enhanced than that of NS-treated control rats. Meanwhile, some artemin was secreted out the smooth muscle cells of dural vasculature (Fig. 5a-f).

Double immunofluorescence showed that low level of GFRα3 was present in the cytoplasm of TG neurons. In addition, an increased number of GFRα3 immunoreactivity neurons in the TG were observed in NTG group compared to the NS control group (Fig. 5j-l).

## Discussion

NTG, an exogenous NO donor, is known to induce migraine-like headache in migraineurs and rodents [18]. Systemic administration of NTG has been reported to cause inflammation and sensitization of primary afferents of the trigeminovascular system [19–21]. In this study, we explored the locations and expression of artemin and its selective receptor GFRα3 in an animal model of migraine, with special attention given to their possible involvement in the pathogenesis of migraine.

In recent years, the role of artemin in mediating neuropathic and inflammatory pain has been paid much attention [8, 22]. The level of artemin is known to be elevated after peripheral inflammation or nerve injury [7, 8]. It has been demonstrated that infusion of NTG lead to vasodilation of the meningeal blood vessels and then modulate the inflammatory factors release [23]. In this study, we found that both the mRNA and protein levels of artemin in the dura mater were up-regulated following NTG administration. In the time course of NTG-induced mRNA and protein expression levels of artemin we found a delayed response, which was consistent with delayed meningeal inflammation of earlier studies. In their reports, migraine attacks do not occur immediately after NTG administration with the vasodilatory effects of the drug, but rather after 2 to 6 h [21, 24]. The trends of the mRNA expression apparently were mismatched with that of protein expression, which might be due to the delayed expression from mRNA to functional receptor protein. In addition, artemin was shown to be located in the smooth muscle cells of dural vasculature, which was in agreement with a study by McIlvried, who firstly reported the expression of artemin in the dura mater of rats [11]. Moreover, in a migraine model, our findings supplied the firsthand evidence that the immunfluorescence intensity of artemin was obviously up-regulated and a certain amount of artemin was secreted out the smooth muscle cells of the dural vascular, indicating that the release of artemin from vasculature may contribute to the dural inflammation of migraine pain.

Artemin exerts its roles in intracellular signaling pathways through the receptor complex of ret and GFRa3, and recent studies suggest that GFRa3 was present in trigeminal afferents that innervate the dura [9, 11]. To further explore the possible roles of artemin in migraine, the alterations in its selective receptor GFRα3 in response to NTG were continually determined. We found that both the mRNA and protein levels of GFRα3 in the TG were substantially up-regulated as a result of the NTG injection. Meanwhile, the number of GFRα3 immunoreactive TG neurons was increased after NTG treatment, indicating that the release of artemin from the vascular smooth muscle cells may activate the peripheral trigeminal nerve via binding GFRα3 in a rat migraine model. Some observations have shown that GFRα3 appeared to be present in similar subpopulations of nociceptive afferents in the TG [10, 25], which suggests that artemin and GFRα3 might play a particularly important role in nociceptive processing in the trigeminal system and consequently contribute to the pathology of migraine.

Previous studies have reported that overexpression of artemin in skin and tongue enhances expression of nociceptor TRPV1 in DRG and TG, thereby leading to behavioral and oral sensitivity [8, 26]. Moreover, activation of TRPV1 has been shown to promote the release of calcitonin gene-related peptide (CGRP) from trigeminal nerve terminals, which contribute to the neurogenic inflammation in the initiation of a migraine attack [27, 28]. However, other studies suggest that TRPV1 has no effect on the pathogenesis of migraine and TRPV1 antagonists are not effective for migraine treatment [29–31]. Thus, the role of the TRPV1 in migraine pain is still controversial to date, and the downstream regulatory mechanisms underlying the actions of artemin and GFRα3 on migraine pain require to be studied further.

## Conclusion

In summary, our study revealed that the enhanced activities of artemin and GFRα3 might be critical processes in migraine pathogenesis and serve as the possible key factor in inducing migraine pain. Taken together, artemin and its selective receptor GFRα3 might be novel targets for therapeutic strategies of migraine.

## Abbreviations

CGRP: Calcitonin gene related peptide
DRG: Dorsal root ganglia
GDNF: Glial cell line-derived neurotrophic factor
GFRα3: GDNF family receptor alpha 3
NO: Nitric oxide
NS: Normal saline
NTG: Nitroglycerin
PBS: Phosphate-buffered solution
qRT-PCR: Quantitative real-time reverse transcription-polymerase chain reaction
TG: Trigeminal ganglia
TRPV1: Transient receptor potential vanilloid 1
α-SMA: α-smooth muscle actin
